# Knowledge, attitude, and practice about e-cigarettes among university students: A comparative study between Afghanistan, the Netherlands, and Turkey

**DOI:** 10.1371/journal.pone.0340344

**Published:** 2026-05-28

**Authors:** Mansoorullah Muradi, Lima Oria, Tariq Aziz Azizi

**Affiliations:** 1 Department of Public Health, Kabul University of Medical Sciences, Kabul, Afghanistan; 2 Medicine, International School of Medicine, Istanbul Medipol University, Istanbul, Turkey; 3 Department of Public Health, Kabul University of Medical Sciences, Kabul, Afghanistan; University of Catania, ITALY

## Abstract

**Background:**

The rise in e-cigarette use among young adults has raised significant public health concerns. Initially introduced as a harm-reduction tool, e-cigarettes have increasingly become a popular alternative to traditional smoking. However, misconceptions about their safety, social acceptability, and long-term health effects persist among university students.

**Methods:**

This study adopts an analytical cross-sectional design and employs a foundational approach by administering an online questionnaire. The study uses a quantitative methodology to explore universities students’ knowledge, attitudes, and perceptions regarding e-cigarettes. Data collection was facilitated through a survey distributed via Google Forms, and statistical analysis was performed using SPSS (Statistical Package for the Social Sciences) 26 to identify significant associations.

**Results:**

The findings revealed that 54.2% of students reported using e-cigarettes, with a higher prevalence among those with close friends or family members who smoked. Knowledge about e-cigarettes was generally low, with 56.2% demonstrating poor understanding, particularly regarding the health risks and regulatory status of e-cigarettes. Attitudes were largely negative, with 73.4% expressing concerns about vaping, though 62.7% believed it was more socially acceptable than traditional smoking. Peer influence was a major factor, as 74.6% of e-cigarette users had close friends who also vaped. A significant association was found between smoking tobacco and e-cigarette use (p = 0.00), indicating a trend of dual-use rather than e-cigarettes serving as a quitting tool.

**Conclusion:**

The study highlights a critical gap in e-cigarette knowledge and a strong influence of social and environmental factors on vaping behaviors. Despite negative attitudes toward vaping, its perceived social acceptability remains high. Public health interventions should focus on targeted educational campaigns, peer-led awareness programs, and regulatory measures to address misconceptions and reduce e-cigarette use among young adults.

## Introduction

Electronic cigarettes (e-cigarettes) are electronic devices that deliver nicotine in aerosol form rather than smoke. [1]

Vaping, a form of electronic smoking, has gained widespread popularity among a diverse range of young and adult users in recent years. In the United States, over 40 million people use vaping products, with about 5 million specifically using e-cigarettes. This trend is not limited to the U.S.; other countries, including the UK, have also seen significant increases in vaping. For example, from 2012 to 2016, vaping use in the UK grew by 60%. The rapid advancement in vaping technology has contributed to its rising acceptance and use as an alternative to traditional cigarettes. [1] In North America, particularly the U.S., vaping consumption has been overgrown since 2014, with 20% of adults aged 18–26 reporting e-cigarette use. In 2014, about 10% of surveyed individuals acknowledged regular vaping, a number that has consistently increased over the years. [2] By 2013, only 2.6% of adults used e-cigarettes regularly. Among younger age groups. For instance, by 2014, around 20% of adults aged 18–26 reported using e-cigarettes, with about 13% of the adult population having tried vaping at least once. In addition, there has been a 70% increase in new vaping users in the U.S. from 2013 onwards. Daily usage among North American adults has also increased, with estimates indicating that about 3.7% regularly vape. Similar trends are observed in Canada and Mexico [3]. In Canada, vaping use grew by 13.2% in 2015, with around 1% using them daily, with 3.2% of adults reporting e-cigarette use within the last month. [4] Despite a ban on e-cigarettes in Mexico since 2013, usage increased by over 50% in one year, indicating strong public interest in vaping over traditional tobacco products [5]. E-cigarette (e-cigarette) use has increased globally over the past decade, particularly among young adults, raising growing public health concerns [6]. Although initially introduced as a harm-reduction alternative for smokers, e-cigarettes are not risk-free and remain under scrutiny by major health authorities, including the World Health Organization (WHO) [7]. Evidence suggests that while some smokers perceive e-cigarettes as less harmful than traditional tobacco, potential risks such as nicotine dependence and respiratory effects persist [8].

A 2024 cross-sectional study conducted among 1002 university students in Palestine examined the prevalence and determinants of e-cigarette use, as well as knowledge, attitudes, and perceptions related to vaping. The study reported that 18.1% of students were current e-cigarette users. Users demonstrated significantly lower knowledge scores regarding the health effects of e-cigarettes compared to non-users. E-cigarette use was strongly associated with traditional cigarette smoking, waterpipe use, and having close social contacts who smoke. Binary logistic regression analysis identified smoking status (participant and mother) and lower knowledge levels as significant predictors of e-cigarette use. The authors concluded that insufficient awareness contributes to higher prevalence among youth and emphasized the need for targeted educational interventions and strengthened tobacco control policies [9]The WHO estimates that tobacco use contributes to more than eight million deaths annually worldwide, and concerns remain that the expanding use of e-cigarettes may further complicate tobacco control efforts, particularly among youth and non-smokers [7,10].

E-cigarettes, commonly known as vapes, are battery-powered devices designed to deliver nicotine through aerosolized vapor. These devices typically consist of a rechargeable battery, a heating element, and a cartridge or tank containing a liquid mixture of nicotine, flavorings, and other chemicals [9,11].

The liquid, also known as “e-liquid” or “vape juice,” generally contains nicotine, propylene glycol, glycerin, and a variety of flavoring agents. [12] Also, which vaporizes a nicotine-containing solution, was marketed as a safer alternative to combustible cigarettes, mainly because it avoids the combustion process that produces harmful tar and other toxic chemicals found in traditional tobacco smoke. [10,13]. When the user activates the device, the heating element vaporizes the liquid, creating an aerosol that is then inhaled into the lungs. [14] E-cigarettes have evolved, with more recent models, such as pod-based systems, delivering significantly higher concentrations of nicotine than earlier devices. These newer devices, particularly popular among young adults, can deliver nicotine levels that rival those found in traditional cigarettes, thus increasing the risk of addiction. [15,16] Notably, the aerosol generated by e-cigarettes contains harmful substances beyond nicotine, including formaldehyde, acetaldehyde, heavy metals like lead and chromium, and other volatile organic compounds, all of which can be inhaled deep into the respiratory system. [9,11] Despite their sleek designs and the marketing of flavored liquids, the safety of these devices remains highly questionable, especially when used over the long term. [16]

The health risks associated with e-cigarette use have been increasingly documented, particularly respiratory and cardiovascular diseases. Chronic exposure to e-cigarette aerosols has been shown to cause airway inflammation, alveolar damage, and ciliary dysfunction, all of which are precursors to conditions such as Chronic Obstructive Pulmonary Disease (COPD) and bronchitis. [12,14] Studies also highlight that the presence of carcinogenic substances, such as formaldehyde and acetaldehyde, elevates the risk of developing cancers, particularly lung cancer. [11] Furthermore, the inhalation of heavy metals like nickel, chromium, and lead can lead to cardiovascular complications, including an increased risk of myocardial infarction and stroke. [14] Neurological impairments have also been linked to e-cigarette use, particularly due to the high concentrations of nicotine found in modern devices. Nicotine is known to interfere with brain development in younger users, leading to cognitive impairments and a heightened risk of addiction. [14,16] Therefore, while e-cigarettes are often marketed as a healthier alternative to traditional smoking, emerging evidence suggests that their long-term use may result in serious, potentially life-threatening diseases. [9,16]

A growing body of research has investigated the usage patterns, knowledge gaps, and risks associated with e-cigarette use. For instance, a cross-sectional study conducted among university students in Austria revealed a significant lack of understanding regarding the dangers of e-cigarettes. Only 10.3% of the students in the study recommended e-cigarettes as a tool for smoking cessation, pointing to widespread misconceptions about their safety. [15] Similarly, a study at Cairo University found that while 83.7% of students were aware of e-cigarettes, many continued to hold incorrect beliefs about their safety. The study reported that 32.4% of students had tried vaping, despite acknowledging the potential risks. [16] In China, research indicated that only 42.6% of university students knew that e-cigarettes contain nicotine, underscoring the significant knowledge gap among young adults about the contents and risks of vaping. [9] Another study conducted in Saudi Arabia showed that 27.7% of health science students regularly used e-cigarettes, a figure significantly higher than the prevalence of conventional cigarette smoking among the same group. [12] These findings emphasize the urgent need for public health interventions, educational programs, and stricter regulations to address the growing use of e-cigarettes and mitigate their potential public health risks. [11]

As this is an observational exploratory study, no formal hypotheses were predefined. The study aimed to describe patterns in KAP rather than test predictive or causal assumptions.

This research will contribute significantly to improving the understanding of e-cigarette use, particularly its health risks and the misconceptions surrounding its safety. By providing insight into the molecular and physiological effects of e-cigarette use, as well as its impact on public health, this study aims to support the development of more effective public health policies and targeted interventions.

## Materials and methods

### Type of study

This research employs an analytical cross-sectional design, utilizing a structured online questionnaire to gather data. The study follows a quantitative methodology aimed at assessing university students’ knowledge, attitudes, and perceptions concerning e-cigarette use. Data were collected via an online survey distributed through Google Forms.

### Study setting

The study was conducted across three universities: Kabul University (Afghanistan), NHL Stenden University (Netherlands), and Istanbul Medipol University (Turkey). Kabul University of Medical Sciences primarily enrolls medical and public health students, offering education focused on clinical and preventive health. Istanbul Medipol University is a private medical-oriented institution with a strong emphasis on health sciences and biomedical research. NHL Stenden University of Applied Sciences focuses on applied learning and professional training, enrolling students from diverse academic backgrounds. These institutional differences may influence students’ health knowledge, perceptions, and behavioral attitudes toward vaping.

According to official reports from the Student Affairs departments of these institutions, the total student population includes 22,137 at Kabul University, 24,000 at NHL Stenden University, and 46,988 at Medipol University. These universities comprise various faculties, both in medical and non-medical fields. Afghanistan, the Netherlands, and Turkey were selected to represent countries with contrasting socioeconomic development, cultural norms, and tobacco control policies. These countries differ substantially in GDP per capita, regulatory frameworks, and public attitudes toward vaping. The Netherlands represents a high-income country with relatively liberal social norms and harm-reduction-oriented tobacco policies, Turkey represents a middle-income country with strong regulatory enforcement, and Afghanistan represents a low-income setting with more conservative cultural attitudes and limited regulatory infrastructure. This comparative design enables an examination of how economic, cultural, and policy environments influence knowledge, attitudes, and practices (KAP) related to e-cigarette use among university students.

### Duration

The data collection spanned over a period of eight weeks, beginning in the first week of November 2024 and concluding by the end of December 2024

### Sampling method

The three countries were selected based on accessibility, population diversity, and relevance to the research objectives.

A quota sampling technique was adopted for participant selection. Using Epi Info (version 3.2.5.0), a sample size was calculated from the total student population of 93,125. Based on a 5% margin of error, a 50% response distribution, and a 95% confidence level, a sample of 383 students was initially determined. Allowing for a 10% non-response rate (30 students), the final sample size was set at 413 participants. The sample was proportionally allocated to each university as follows: Kabul University – 23.7% (98 students), NHL Stenden University – 50.4% (208 students), and Medipol University – 25.9% (107 students). The questionnaire was then distributed digitally to students from the respective universities. Although quota sampling was used to ensure balanced representation across demographic groups, the sample may not fully represent the general population. This limitation is acknowledged in the discussion.

### Participants

The study focused on both male and female students from medical and non-medical faculties at the selected universities. All participants were selected according to the quota sample and were accessible for inclusion. In total, 413 students completed the questionnaire, achieving the expected sample size.

### Sample size

Using Epi Info version 3.5.2.0, and based on a total population of 93,125 students, the required sample size was calculated with an expected frequency of 50% and a 5% margin of error. This yielded an initial sample of 383 students, increased to 413 after accounting for an anticipated 10% non-response rate.

### Data source and measurement

Participants included male and female students from various faculties. All selected individuals completed the questionnaire, achieving the target sample size.

The KAP questionnaire was adapted from previously published instruments identified through PubMed. underwent content review by public health experts and was pilot-tested among a small group of students to ensure clarity and cultural relevance. The tool Internal consistency was not assessed, as this was an exploratory study. Minor wording adjustments were made to improve comprehension among Afghan participants.

The questionnaire, adapted from PubMed-based tools [17,18], included four sections:

1. Demographic Information – Reporting frequencies and percentages.
2. Knowledge of E-cigarettes – Assessed via 15 binary (true/false) items. A 75% accuracy threshold (at least 11 correct responses) was used to classify participants as having good knowledge, while those scoring below 75% were considered to have poor knowledge.
3. Attitudes Toward E-cigarettes – Included 13 items measured on a binary agree/disagree scale. Positive attitudes were identified by scores of 75% (9) or higher. whereas those scoring below 75% were considered to have a negative attitude. The statements reflected health-related perceptions of e-cigarette use.
4. Practices Regarding E-cigarettes – Evaluated through 13 items examining use patterns, frequency, purchasing behavior, and duration of use. Current users were scored as ‘0’, and non-users or former users were scored as ‘1’.

### Statistical methods

Upon completion of data collection, responses were entered into SPSS version 26 for processing. Prior to analysis, the dataset was reviewed to ensure accuracy and address any inconsistencies. Data analysis was conducted using SPSS software, and visual representations were generated accordingly. Descriptive statistics, including frequencies and percentages, were used to summarize participant characteristics. Associations between categorical variables were assessed using the Chi-square test. A p-value of less than 0.05 was considered statistically significant.

### Ethical consideration

I hereby declare that this research titled “Knowledge, Attitude, and Practice about E-cigarettes among University Students: A Comparative Study between Afghanistan, Netherlands, and Turkey” has been reviewed and approved by the Research Ethics Committee of Khatam Al Nabieen University. The study complies with the ethical principles and standards for conducting medical research in Afghanistan.

- Approval Code: AF, knu.edu.af.rec 02
- Approval Date: September 10, 2023

The approval was granted based on the documents submitted to the committee on August 10, 2023. The Principal Investigator bears full responsibility for ensuring that all legal and professional obligations are fulfilled throughout the research process. Written informed consent was obtained from each participant prior to their inclusion in the study. The confidentiality and anonymity of participants were strictly maintained throughout the research process. All ethical standards outlined in the Declaration of Helsinki were carefully followed.

Note: In Turkey and the Netherlands, anonymous online surveys among adult university students (≥18 years) do not require formal ethics approval under local regulations. Therefore, no additional approval was sought.

### Consent to participate declaration

All individuals who took part in this study were fully informed about the study’s objectives, procedures, and their rights as participants. Participation was entirely voluntary, and written informed consent was obtained from each participant prior to their inclusion in the research.

The informed consent text used for the participants in this study is as follows:

This study is designed as a comparative cross-sectional analysis, meaning that the research evaluates responses from a structured questionnaire. The questionnaire was developed specifically for this study and is organized into four sections, containing 50 questions covering demographic information, and assessing knowledge, attitudes, and practices related to e-cigarettes.

Before giving your consent, please consider the following:

- This study involves no physical or psychological harm to participants or researchers.
- Participation in this study is entirely voluntary.
- The identity of participants has no impact on the study’s outcomes, and therefore, your name will remain confidential and will not be mentioned in the research.
- The findings of this study can be made available to you upon request, following the completion of all necessary legal procedures.
- For any questions or concerns, you may contact “Mansoorullah Muradi” at mansoormuradi79@gmail.com or “Lima Oria” at laimaori11@gmail.com.

We kindly request that you respond honestly to all questions, as this will help ensure the transparency and accuracy of the information collected.

By reading and acknowledging this information, you indicate your willingness to voluntarily participate in this study. You agree to allow the researchers to use the information you provide for research purposes.

## Results

### Demographic characteristics

A total of 413 university students participated in the study, including 210 males (50.8%), 200 females (48.4%), and 3 individuals (0.7%) who preferred not to disclose their gender. Of these, 295 (71.4%) were non-medical students and 118 (28.6%) were from medical faculties. Most respondents were aged 20 years.

Tobacco use was most prevalent among Dutch students (61.5%, n = 123), followed by Afghan (26.5%, n = 53) and Turkish students (12.0%, n = 24). Cohabitation with smokers was reported by 60.0% of Dutch students (n = 132), compared to 21.8% of Afghans and 18.2% of Turkish students. Additionally, 60.4% of Dutch students reported having a family member who used e-cigarettes.

Paternal education was highest among those with tertiary education (63.2%), while 21.8% of mothers had completed only primary school or less. Tertiary education among mothers was most common in Netharlands (44.8%) and least in Afghanistan (12.3%).

E-cigarette use among close friends was acknowledged by 53.4% of Dutch students, 24.7% of Afghan students, and 21.9% of Turkish students. This demographic profile highlights a diverse sample across the three countries, with notable differences in gender distribution and parental education, which may serve as potential confounding factors.

A full comparative breakdown is provided in Table 1.

**Table 1:** Demographic breakdown of all three countries.

Variables	Countries			
**Afghanistan**	**Netherlands**	**Turkey**	**Total**
**Sex**				
Male	56(26.7%)	118(56.2%)	36(17.1%)	210(100%)
Female	42(21.0%)	88(44.0%)	70(35.0%)	200(100.0%)
Prefer not to say	0(0%)	3(100%)	0(0%)	3(100.0%)
**Age**				
<20 years	29(20.6%)	76(53.9%)	36(25.5%)	141(100.0%)
> 20 Years	30(23.6%)	57(44.9%)	40(31.5%)	127(100.0%)
20 years	39(26.9%)	75(51.7%)	31(21.4%)	145(100.0%)
**Major**				
Medical University	9(7.6%)	14(11.9%)	95(80.5%)	118(100.0%)
Non-Medical University	89(30.2%)	194(65.8%)	12(4.1%)	295(100.0%)
**Year in University**				
First-Second Year	46(18.9%)	127(52.3%)	70(28.8%)	243(100.0%)
Third-Fourth Year	52(30.6%)	81(47.6%)	37(21.8%)	170(100.0%)
**Living area**				
Urban	83(27.1%)	133(43.5%)	90(29.4%)	306(100.0%)
Rural	15(14.0%)	75(70.1%)	17(15.9%)	107(100.0%)
**Father’s educational level**				
primary or less	23(47.9%)	23(47.9%)	2(4.2%)	48(100.0%)
Secondary education (high school or equivalent)	29(27.9%)	55(52.9%)	20(19.2%)	104(100.0%)
Tertiary education (college, university, or higher)	46(17.6%)	130(49.8%)	85(32.6%)	261(100.0%)
**Mother’s educational level**				
primary or less	40(44.4%)	40(44.4%)	10(11.1%)	90(100.0%)
Secondary education (high school or equivalent)	38(23.8%)	95(59.4%)	27(16.9%)	160(100.0%)
Tertiary education (college, university, or higher)	20(12.3%)	73(44.8%)	70(42.9%)	163(100.0%)
**Monthly living cost (USD)**				
≤300	77(34.1%)	89(39.4%)	60(26.5%)	226(100.0%)
>300	21(11.2%)	119(63.6%)	47(25.1%)	187(100.0%)
**Smoking tobacco cigarette**				
Yes	53(26.5%)	123(61.5%)	24(12.0%)	200(100.0%)
No	45(21.1%)	85(39.9%)	83(39.0%)	213(100.0%)
**Currently live with smokers.**				
Yes	48(21.8%)	132(60.0%)	40(18.2%)	220(100.0%)
No	50(25.9%)	76(39.4%)	67(34.7%)	193(100.0%)
**Family members ever use tobacco cigarettes.**				
Yes	53(24.0%)	121(54.8%)	47(21.3%)	221(100.0%)
No	45(23.4%)	87(45.3%)	60(31.3%)	192(100.0%)
**Family members ever use e-cigarettes**				
Yes	41(22.5%)	110(60.4%)	31(17.0%)	182(100.0%)
No	57(24.7%)	98(42.4%)	76(32.9%)	231(100.0%)
**Close friends ever use tobacco cigarettes.**				
Yes	61(24.2%)	136(54.0%)	55(21.8%)	252(100.0%)
No	37(23.0%)	72(44.7%)	52(32.3%)	161(100.0%)
**Close friends ever use e-cigarettes (vaping)**				
Yes	62(24.7%)	134(53.4%)	55(21.9%)	251(100.0%)
No	36(22.2%)	74(45.7%)	52(32.1%)	162(100.0%)

92.3% of the study population has heard of ECs mostly through, university/school, social media, and friends (30.9%, 23.2% 21.9% respectively).

### E-cigarette knowledge

Knowledge gaps were evident among participants across key areas. Notably, 64.6% incorrectly believed that e-cigarettes are approved by the U.S. Food and Drug Administration (FDA), and 51.3% falsely assumed they do not contribute to secondhand smoke exposure. In contrast, 70.5% correctly identified that flavorings differ in harmfulness.

Knowledge regarding health risks was moderately strong: 66.8% recognized the link to bladder cancer, 56.2% acknowledged the association with lung cancer, and 73.8% understood potential impairment of heart and lung function due to e-cigarette use.

These results indicate a basic awareness of some health risks associated with vaping; however, significant misconceptions persist, particularly concerning regulatory approval and secondhand exposure.

A full distribution of responses is provided in Table 2.

**Table 2:** E-cigarette knowledge questions frequency and percentage.

Knowledge questions		No.	Valid %
1	**E-cigarettes are associated with bladder cancer.**		
	False__Wrong Answer	137	33.2%
	True__Correct Answer	276	66.8%
2	**E-cigarettes are FDA Approved**		
	True__ Wrong Answer	267	64.6%
	False__ Correct Answer	146	35.4%
3	**Some flavors of E-cigarettes are more harmful than others.**		
	False__ Wrong Answer	122	29.5%
	True__ Correct Answer	291	70.5%
4	**Swallowing the liquid in E-cigarettes accidentally can cause poisoning that is potentially fatal.**		
	False__ Wrong Answer	96	23.2%
	True__ Correct Answer	317	76.8%
5	**E-cigarettes are not associated with lung cancer.**		
	True__ Wrong Answer	181	43.8%
	False__ Correct Answer	232	56.2%
6	**E-cigarettes impair lung and heart functions.**		
	False__ Wrong Answer	108	26.2%
	True__ Correct Answer	305	73.8%
7	**E-cigarettes are not contributed to secondhand smoking**		
	True__ Wrong Answer	212	51.3%
	False__ Correct Answer	201	48.7%
8	**E-cigarettes can have an effect on fetal development**		
	False__ Wrong Answer	85	20.6%
	True__ Correct Answer	328	79.4%
9	**Nicotine is present in most E-cigarettes**		
	False__ Wrong Answer	87	21.1%
	True__ Correct Answer	326	78.9%
10	**Harmful flavorings and toxins are found in the E-cigarette aerosol**		
	False__ Wrong Answer	76	18.4%
	True__ Correct Answer	337	81.6%
11	**E-cigarettes are harmful**		
	True__ Wrong Answer	178	43.1%
	False__ Correct Answer	235	56.9%
12	**Some components of the liquid found in E-cigarettes can cause harmful lung conditions**		
	False__ Wrong Answer	93	22.5%
	True__ Correct Answer	320	77.5%
13	**E-cigarettes are not addictive**		
	True__ Wrong Answer	163	39.5%
	False__ Correct Answer	250	60.5%
14	**E-cigarettes are suitable for pregnant women**		
	True__ Wrong Answer	137	33.2%
	False__ Correct Answer	276	66.8%
15	**E-cigarettes are suitable for children**		
	True__ Wrong Answer	141	34.1%
	False__ Correct Answer	272	65.9%

### E-cigarette Attitude

Participants’ attitudinal responses reflected mixed views on e-cigarette use. A total of 62.7% perceived vaping as more socially acceptable than traditional smoking, while 48.9% considered recreational experimentation acceptable. Additionally, 55.4% viewed e-cigarettes as effective cessation tools; 59.8% believed they assist in reducing or quitting smoking, and 54.5% supported their use as substitutes for conventional cigarettes.

The full distribution of responses is presented in Table 3

**Table 3:** E-cigarette attitude questions frequency and percentage.

Attitude questions		No.	Valid %
1	**Should E-cigarettes be recommended to a non-smoker?**		
	No__ Negative Attitude	217	52.5%
	Yes__ Positive Attitude	196	47.5%
2	**Do you think E-cigarettes are harmful to health?**		
	Yes__ Negative Attitude	308	74.6%
	No__ Positive Attitude	105	25.4%
3	**Should the use of E-cigarettes be allowed in places that do not allow smoking?**		
	No__ Negative Attitude	229	55.4%
	Yes__ Positive Attitude	184	44.6%
4	**Would you consider someone who uses E-cigarettes a smoker?**		
	Yes__ Negative Attitude	276	66.85
	No__ Positive Attitude	137	33.2%
5	**Do you think the use of E-cigarettes can lead to reliance?**		
	Yes__ Negative Attitude	275	66.6%
	No__ Positive Attitude	138	33.4%
6	**Do you think the government should regulate the use of E-cigarettes?**		
	Yes__ Negative Attitude	267	64.6%
	No__ Positive Attitude	146	35.4%
7	**Do you feel more comfortable using or openly talking about smoking E-cigarettes, compared to cigarettes?**		
	No__ Negative Attitude	158	38.3%
	Yes__ Positive Attitude	255	61.7%
8	**Do you feel it is more socially acceptable to smoke E-cigarettes, compared to cigarettes?**		
	No__ Negative Attitude	154	37.3%
	Yes__ Positive Attitude	259	62.7%
9	**Should E-cigarettes be used as a replacement for regular cigarettes?**		
	No__ Negative Attitude	188	45.5%
	Yes__ Positive Attitude	225	54.5%
10	**Experiment with E-cigarettes for pleasure?**		
	No__ Negative Attitude	211	51.1%
	Yes__ Positive Attitude	202	48.9%
11	**Do you think using E-cigarettes would be an effective way to help in smoking cessation?**		
	No__ Negative Attitude	184	44.6%
	Yes__ Positive Attitude	229	55.4%
12	**Do you think it is acceptable to use E-cigarettes as a smoking cessation method?**		
	No__ Negative Attitude	200	48.4%
	Yes__ Positive Attitude	213	51.6%
13	**Do you think E-cigarettes can help people cut down on cigarettes or quit smoking?**		
	No__ Negative Attitude	166	40.2%
	Yes__ Positive Attitude	247	59.8%

### E-cigarette practice

The prevalence of e-cigarette use was 54.2%. Among users, 27.1% vaped 1–2 days in the previous week, and 6.3% reported daily use exceeding 20 times. Authorized retailers supplied devices for 41.2% of users, and 38.5% found access to products easy. Most spent 10–28 EUR per purchase (29.8%). Initial use generally began before age 18, with 39.5% having no intention to quit vaping.

Detailed information regarding these findings is presented in Table 4.

**Table 4:** E-cigarette practice question frequency and percentage.

Practice questions		No.	Valid %
1	**Currently using e-cigarettes**		
	Yes	224	54.2%
	No	189	45.8%
2	**If yes, Duration of use within the last 7 days**		
	> 5 days	86	20.8%
	3–4 days	26	6.3%
	1–2 days	112	27.1%
3	**Frequencies of e-cigarette usage per day**		
	<20 times a day	103	24.9%
	>20 times a day	26	6.3%
	Not Daily	95	23.0%
4	**Time of initiating usage after waking up**		
	After 1–2 hours	97	23.5%
	It varies	94	22.8%
	Immediately after waking-up	33	8.0%
5	**Place of purchase e-cigarette**		
	Authentic store	170	41.2%
	Online shop	54	13.1%
6	**Ease of purchase?**		
	Yes	159	38.5%
	No	65	15.7%
7	**Price of per purchase (EUR)**		
	>28	10	2.4%
	≥10	91	22.0%
	10─28	123	29.8%
8	**Price of e-cigarette liquid (EUR)**		
	< 5	21	5.1%
	>4	87	21.1%
	4─5	116	28.1%
9	**Age of the first trail**		
	< 18 years old	155	37.5%
	≥18 years old	69	16.7%
10	**Intention to quit**		
	Yes	164	39.7%
	No	60	14.5%

This study provides valuable insights into the factors influencing e-cigarette use, particularly its association with traditional smoking habits, social and family influence, and demographic characteristics. Additionally, the findings highlight significant patterns in knowledge, attitudes, and practices KAP (Knowledge Attitude and Practice) related to smoking and vaping. The statistically significant p-values (0.00) across multiple variables indicate strong associations, underscoring the impact of environmental, social, and educational factors on smoking behavior.

Of the participants, 56.2% (n = 232) demonstrated poor knowledge of e-cigarettes. Turkish students showed higher knowledge levels (43.1%) compared to those from Afghanistan (21.0%) and the Netherlands (35.9%) (p = 0.00). Attitudes were predominantly negative, with 73.4% holding poor attitudes and only 26.6% positive.

Regarding practice, 54.2% exhibited good practices, while 45.8% had poor ones. The Netherlands showed the highest prevalence of poor knowledge (61.6%) and the highest proportion of positive attitudes (62.7%). Conversely, Turkey and Afghanistan reported lower positive attitudes (12.7% and 24.5%, respectively) and higher poor practice rates, especially in Turkey (44.4%) (p = 0.00).

Sex was significantly associated with knowledge (p = 0.001); females (59.1%) had better knowledge than males (40.3%). Attitudes did not differ significantly by sex (p = 0.2). However, practice varied (p = 0.001), with females demonstrating better practices (59.4%) than males (40.2%).

These findings highlight notable gaps in knowledge and generally negative attitudes despite moderate use practices, emphasizing the need for targeted educational interventions.

For further details, please refer to Table 5 below

**Table 5:** Knowledge, Attitude and Practice.

Variables	Frequency (n)			Valid%			
**Good knowledge**	181			43.8%			
**Poor knowledge**	232			56.2%			
**Good Attitude**	110			26.6%			
**Poor Attitude**	303			73.4%			
**Good Practice**	224			54.2%			
**Poor Practice**	189			45.8%			
**Variables**	**Good Practice**	**Poor Practice**		**Total**			**P- value**
**Good knowledge**	46(25.4%)	135(74.6%)		181(100.0%)			0.00
**Poor knowledge**	178(76.7%)	54(23.3%)		232(100.0%)			
**Variables**	**Countries**						
	**Afghanistan**	**Netherlands**	**Turkey**			**Total**	**P- value**
**Good knowledge**	38(21.0%)	65(35.9%)	78(43.1%)			181(100.0%)	0.00
**Poor knowledge**	60(25.9%)	143(61.6%)	29(12.5%)			232(100.0%)	
**Good Attitude**	27(24.5%)	69(62.7%)	14(12.7%)			110(100.0%)	
**Poor Attitude**	71(23.4%)	139(45.9%)	93(30.7%)			330(100.0%)	
**Good Practice**	54(24.1%)	147(65.6%)	23(10.3%)			224(100.0%)	
**Poor Practice**	44(23.3%)	61(32.3%)	84(44.4%)			189(100.0%)	
**Variables**	**Sex**						
	**Male**	**Female**	**Prefer not to say**		**Total**		**P- value**
**Good knowledge**	73(40.3%)	107(59.1%)	1(0.6%)		181(100.0%)		0.001
**Poor knowledge**	137(59.1%)	93(40.1%)	2(0.9%)		232(100.0%)		
**Good Attitude**	58(52.7%)	50(45.5%)	2(1.8%)		110(100.0%)		0.2
**Poor Attitude**	150(49.5%)	152(50.2%)	1(0.3%)		303(100.0%)		
**Good Practice**	90(40.2%)	133(59.4%)	1(0.4%)		224(100.0%)		0.001
**Poor Practice**	110(58.2%)	77(40.7%)	2(1.1%)		189(100.0%)		

The analysis revealed significant associations (p = 0.00) between tobacco cigarette smoking and e-cigarette use. Among current e-cigarette users, 72.8% also smoked tobacco, whereas only 19.6% of non-e-cigarette users smoked tobacco. Living with smokers was significantly linked to e-cigarette use, with 69.2% of users cohabiting with smokers. Similarly, 65.2% of e-cigarette users had family members who smoked tobacco, compared to 39.7% of non-users. Having family members who vape was also significantly associated; 65.2% of users reported family vaping versus 22.2% among non-users. Peer influence showed strong correlations: 74.6% of e-cigarette users had close friends who vaped, and 74.1% had friends who smoked tobacco, compared to 44.4% and lower rates among non-users respectively. These findings underscore familial and social environments as key factors influencing vaping behavior.

**Table:** 

Currently using e-cigarettes	Smoking tobacco cigarette			
**yes**	**No**	**Total**	**P- value**
**yes**	163(72.8%)	61(27.2%)	224(100.0%)	**0.00**
**No**	37(19.6%)	152(80.4%)	189(100.0%)	
**Currently using e-cigarettes**	**Currently live with smokers**			
	**yes**	**No**	**Total**	**P- value**
**yes**	155(69.2%)	69(30.8%)	224(100.0%)	**0.00**
**No**	65(34.4%)	124(65.6%)	189(100.0%)	
**Currently using e-cigarettes**	**Family members ever use tobacco cigarettes**			
	**yes**	**No**	**Total**	**P- value**
**yes**	146(65.2%)	78(34.8%)	224(100.0%)	**0.00**
**No**	75(39.7%)	114(60.3%)	189(100.0%)	
**Currently using e-cigarettes**	**Family members ever use e-cigarettes (vaping)**			
	**yes**	**No**	**Total**	**P- value**
**yes**	140(62.5%)	84(37.5%)	224(100.0%)	**0.00**
**No**	42(22.2%)	147(77.8%)	189(100.0%)	
**Currently using e-cigarettes**	**Close friends ever use e-cigarettes (vaping)**			
	**yes**	**No**	**Total**	**P- value**
**yes**	167(74.6%)	57(25.4%)	224(100.0%)	**0.00**
**No**	84(44.4%)	105(55.6%)	189(100.0%)	
**Currently using e-cigarettes**	**Close friends ever use tobacco cigarettes**			
	**yes**	**No**	**Total**	**P- value**
**yes**	166(74.1%)	58(25.9%)	224(100.0%)	**0.00**
**No**	86(45.5%)	103(54.5%)	189(100.0%)	

## Discussion

This study assessed the Knowledge, Attitudes, and Practices (KAP) regarding e-cigarettes among university students in Afghanistan, the Netherlands, and Turkey. Observed cross-country differences may reflect broader sociocultural, economic, and regulatory influences. Conservative cultural and religious norms in Afghanistan may discourage vaping behavior, while higher disposable income and greater product accessibility in the Netherlands may facilitate experimentation. Turkey’s relatively strict regulatory enforcement may contribute to moderated usage patterns. These contextual factors provide a more comprehensive explanation beyond peer influence alone.

To provide a broader context, we compare our findings with studies conducted in Saudi Arabia. [12], China [9], Indonesia [19], Egypt [16], Jordan [20], Malaysia [21] and France [17]. The findings highlight significant variations in e-cigarette awareness, attitudes, and usage trends across different cultural and regulatory environments.

Our study found that e-cigarette use varied significantly based on demographic factors such as age, gender, and academic background. In Afghanistan, the Netherlands, and Turkey, the majority of e-cigarette users were male students aged 19–25. This aligns with findings from Saudi Arabia, where 66.2% of e-cigarette users were male, and most users were between 19 and 22 years old [12].

In contrast, studies from France and China showed that while male students were more likely to use e-cigarettes, female users were also prevalent. [9,17].

Financial status also played a role in e-cigarette consumption. In our study, students from middle- to high-income families reported higher e-cigarette use, which is consistent with findings from France, where students facing fewer financial difficulties were more likely to experiment with vaping. [17].

Our findings are consistent with recent evidence from Palestine, where a cross-sectional study among university students reported an e-cigarette prevalence of 18.1% and identified lower knowledge levels as a significant determinant of use, highlighting the importance of educational interventions to reduce vaping among young adults. [9]

Demographic factors such as age, gender, and financial status conditions play a significant role in shaping e-cigarette consumption patterns among university students. While young adult males remain the predominant users, social influences and economic background also contribute to e-cigarette adoption. Future research should explore the long-term health impacts of e-cigarettes across diverse demographic groups, with a focus on mental health interventions and policy regulation to reduce vaping among at-risk populations.

### Knowledge of E-Cigarettes

Our study found that Turkish students exhibited the highest level of awareness (43.1%), followed by Afghan (21.0%) and Dutch students (35,9%). This disparity is consistent with findings from China, where students demonstrated moderate awareness but had significant gaps in understanding the nicotine content and long-term health risks. [9]

The study reported that 10.5% of Jordanians. [20] Students used e-cigarettes, whereas our study found a significantly higher prevalence (54.2%). This stark difference suggests that cultural, social, and regulatory factors may influence e-cigarette adoption. The higher prevalence in the international sample, particularly in the Netherlands, could be attributed to greater social acceptance and easier access to e-cigarettes compared to Jordan. [20]. These misconceptions reflect a global issue: students often receive information from social media, friends, and advertisements, which may not provide accurate health information. The study found that medical students in Jordan had better knowledge than non-medical students, a pattern also observed in our study, where Turkish students many of whom were medical students had higher knowledge levels compared to Afghan and Dutch students.

### Attitudes toward E-Cigarettes

Attitudes toward e-cigarettes varied significantly across different countries. In our study, Afghan and Turkish students held predominantly negative views (23.4%) (30.7%), whereas Dutch students were more accepting (62.7% positive attitude).

The studies in Indonesia show that students’ attitudes toward e-cigarettes are influenced by their social environment. The study revealed that many Jordanian students perceived e-cigarettes as a less harmful and less addictive alternative to traditional cigarettes. Similarly, in our study, 62.7% felt e-cigarettes were more socially acceptable than traditional cigarettes, and 54.5% believed e-cigarettes should replace regular cigarettes. However, both studies also indicate a significant portion of students hold negative attitudes toward e-cigarettes, with 73.4% of international students expressing concerns about their safety [19].

In Malaysia, strong opposition was observed, with 62.4% of students supporting a ban on e-cigarettes. [21]. Similarly, in Saudi Arabia, although some students saw e-cigarettes as a smoking cessation tool, many viewed them as equally addictive as traditional cigarettes. [12].

Conversely, Indonesian students displayed highly favorable perceptions, with 91.3% expressing positive attitudes. [19].

These findings indicate that cultural beliefs, policy regulations, and exposure to e-cigarette users significantly shape students’ attitudes toward vaping.

### E-Cigarette use and prevalence

The prevalence of e-cigarette use among university students showed notable differences across countries. In our study, Turkish students reported the highest e-cigarette use (44.4%), followed by Afghan (23.3%) and Dutch students (32.3%).

In Saudi Arabia, 27.7% of health science students reported using e-cigarettes, a rate significantly higher than conventional cigarette smoking (14.1%) [12]. France had a similar prevalence, with 23.0% of students having tried e-cigarettes. [17].

Malaysia reported a relatively low prevalence (6.6%) [21]while in Egypt, 7.3% of medical students reported usage, with a higher prevalence among males [16]. Jordan had a significantly higher prevalence (37.4%) [20], whereas China and Indonesia reported lower usage rates (8.2%) [19]

A consistent finding across all studies was the strong influence of peer networks on e-cigarette use. Students who had friends or family members using e-cigarettes were more likely to experiment with vaping. This trend was evident in Saudi Arabia and Malaysia, where students with vaping peers had significantly higher usage rates. [12,21].

### E-Cigarettes as a smoking cessation tool

In our study, social media (23.1%) and University/Schools (30.9%) were the most common sources of information on e-cigarettes but in Jordan, social media (43.1%) and friends (32.9%) were the most common sources of information on e-cigarettes. Additionally, both studies found a strong correlation between conventional cigarette smoking and e-cigarette use, reinforcing the idea that e-cigarettes are not always used as a smoking cessation tool but rather as an additional form of nicotine consumption.

Countries with strict regulations, such as Malaysia and Jordan, exhibited lower e-cigarette prevalence rates. In Malaysia, the National Fatwa Council declared vaping forbidden (haram), significantly influencing students’ attitudes and behaviors. [20,21]. Similarly, Jordan prohibits e-cigarette advertising and sales to individuals under 19 [20].

By contrast, Saudi Arabia and Indonesia, where regulations are more lenient, reported higher e-cigarette usage rates. [12,19]. France, despite implementing regulations restricting sales to minors, still had a high proportion of experimental users. [17].

These findings emphasize the impact of government policies and public health campaigns on vaping behaviors. Countries with stronger tobacco control measures tend to see lower e-cigarette usage among youth. Overall, peer influence, social media exposure, and regulatory environments play crucial roles in shaping students’ e-cigarette knowledge, attitudes, and behaviors. Future public health efforts should focus on harmonizing policies and increasing educational initiatives to mitigate the growing trend of e-cigarette use among university students worldwide.

Differences in KAP outcomes may be partially explained by variation in national tobacco control strategies under the WHO MPOWER framework. The Netherlands has implemented comprehensive smoke-free policies and advertising restrictions, Turkey has strengthened cessation support and enforcement mechanisms, whereas Afghanistan has limited implementation of MPOWER measures due to regulatory and resource constraints. These disparities may influence awareness levels, accessibility, and public perceptions of e-cigarette use.

This study has several limitations that should be considered. First, the use of online questionnaire may have introduced slection bias, as participationwas limited to students with internet access and those more willing to respond to online surveys and the data were collected through an online self-administered questionnaire using Google Forms, which may introduce response bias and limit control over who participated. Second, the use of convenience sampling through voluntary participation may affect the representativeness of the sample and reduce the generalizability of the findings. Third, since the study was conducted only among university students in three countries (Afghanistan, Turkey, and the Netherlands), the results may not be applicable to the general population or to students in other regions. Additionally, due to the cross-sectional design of the study, causal relationships between knowledge, attitudes, and practices toward e-cigarettes cannot be established. Despite these limitations, the findings provide valuable insights into the awareness and behavior of university students regarding e-cigarette use across diverse cultural contexts.

## Conclusions

### Key takeaways

E-cigarette use is strongly linked to traditional smoking habits, indicating a potential dual-use problem rather than e-cigarettes serving as an alternative to quitting.

Household smoking environments significantly influence e-cigarette use, reinforcing the need for family-based prevention programs.

Peer influence is one of the most powerful predictors of smoking behavior, making peer-led interventions critical in tobacco control strategies.

Despite moderate knowledge levels, attitudes toward smoking remain poor, suggesting that awareness alone is insufficient—interventions must address social and behavioral aspects.

Cultural, gender, and educational differences play a role in smoking behavior, requiring customized public health approaches based on demographic characteristics.

### Policy and Intervention Recommendations

Policy and Intervention Suggestions:

Targeted Awareness Campaigns: Programs should challenge behaviors and focus on peer groups.

Family-Based Interventions: Smoke-free home initiatives and parental education to reduce youth vaping.

Peer-Led Prevention: Schools and universities should adopt peer-led anti-smoking programs.

Regulations: Stricter e-cigarette sales controls and environmental policies (e.g., public bans) are needed.

Gender-Specific Strategies: Female-focused programs to reinforce avoidance; male-targeted campaigns emphasizing health risks.

Culturally Adapted Approaches: Prevention must consider cultural norms and smoking prevalence differences.

## Supporting information

S1 Data Data Clean. (XLSX)
